# The Effect of Antihypertensive Drugs on Endothelial Function as Assessed by Flow-Mediated Vasodilation in Hypertensive Patients

**DOI:** 10.1155/2012/453264

**Published:** 2012-02-29

**Authors:** Michiaki Miyamoto, Kazuhiko Kotani, Shun Ishibashi, Nobuyuki Taniguchi

**Affiliations:** ^1^Department of Clinical Laboratory Medicine, Jichi Medical University, 3311-1 Yakushiji, Shimotsuke-City, Tochigi 329-0498, Japan; ^2^Division of Endocrinology and Metabolism, Department of Medicine, Jichi Medical University, 3311-1 Yakushiji, Shimotsuke-City, Tochigi 329-0498, Japan

## Abstract

Endothelial dysfunction is found in hypertensive patients and may serve as a prognostic marker of future cardiovascular events. Endothelial function can be assessed noninvasively by flow-mediated vasodilation (FMD). The goal of this paper is to summarize comprehensively the clinical trials that investigated the effects of antihypertensive drugs on endothelial function assessed by FMD in hypertensive patients. A PubMed-based search found 38 clinical trial papers published from January 1999 to June 2011. Significant improvement of FMD after antihypertensive treatment was shown in 43 of 71 interventions (among 38 clinical trial papers). Angiotensin II receptor blockers and angiotensin converting enzyme inhibitors appeared to improve FMD more than other drug types. Antihypertensive treatment can improve endothelial dysfunction when assessed by FMD, although there are conflicting data that require further research.

## 1. Introduction

Atherosclerotic risk factors such as hypertension (HTN), diabetes mellitus, dyslipidemia, obesity, and smoking cause endothelial dysfunction [1–5]. Endothelial dysfunction occurs in the early stages of atherosclerosis and is involved in disease progression as well as the morbid cardiovascular events that often occur in advanced stages of the diseases [1–5]. The endothelium is involved in the control of the coagulation/fibrinolytic system, platelet aggregation, adhesion of leukocytes, and smooth muscle cell proliferation and is important in the maintenance of vascular tone [1, 3]. The response-to-injury hypothesis, proposed by Russell Ross [6], states that atherosclerosis is due to an inflammatory reaction in response to endothelial injury or dysfunction and is supported by numerous basic and clinical investigations [1, 3].

The evaluation of endothelial function is available as a predictor of cardiovascular events and as a surrogate marker for early atherosclerosis [1–3, 7, 8]. There are several methods to evaluate endothelial function that include an invasive method using endothelium-dependent vasodilators injected into a coronary or peripheral artery [7], and flow-mediated vasodilation (FMD), a noninvasive method based on endothelium-dependent arterial vasodilation [9, 10]. FMD was first reported in 1992 by Celermajer et al., as an innovative method of detecting endothelial dysfunction [10]. The sudden release of an artery after transient occlusion causes an increase in shear stress on the vessel wall due to hyperemia and this stimulates endothelial cells to release various physiologically active substances. Nitric oxide (NO) is one of the main substances released by the endothelium and causes relaxation of vascular smooth muscle with a subsequent increase in vascular diameter [1]. FMD is measured from the expansion rate of arterial diameter during the postischemic hyperemia response. Since guidelines for the measurement of FMD have been established [9] and the measuring equipment has been improved, FMD is gaining acceptance as a simple, safe, and valuable method to evaluate endothelial function in clinical practice.

HTN is prevalent worldwide and one of the most important risk factors for atherosclerotic disease [11, 12]. The relationship between FMD and blood pressure has been reported in a general population [13, 14]. FMD was inversely related to age, male gender, systolic blood pressure, body mass index, and smoking in the Framingham study [13]. It was inversely correlated with male gender, blood pressure, glucose, and directly with high-density lipoprotein cholesterol, C-reactive protein, and body mass index in healthy young adults (Young Finns study) [14]. Moreover, treatment of HTN leads to the prevention of atherosclerotic disease [11, 12]. There are several types of antihypertensive drugs used to treat HTN, and several studies investigated the effect of antihypertensive medications on FMD. It is possible that the choice of the best drug to treat HTN in the future could be based on the magnitude of the improvement in endothelial function. This paper comprehensively summarizes the current knowledge from the clinical trials that have evaluated the effect of antihypertensive drugs on FMD in patients with HTN.

## 2. Methodology of FMD

Guidelines for the ultrasound assessment of endothelial-dependent FMD of the brachial artery have been established [9]. There are still some issues with the reproducibility and objectivity of FMD measurements, because it is necessary to record very small changes in vascular diameter [9], and sonographers must receive adequate training and gain experience before they become competent with this technique. Subjects should fast for at least 8 to 12 hours before the measurement. In addition, subjects should not exercise, should not ingest substances that might affect FMD, such as caffeine, or use tobacco for at least 4 to 6 hours before the measurement.

FMD is assessed in a subject's right arm in the supine position in a quiet, temperature-controlled room. The brachial artery is imaged above the antecubital fossa in the longitudinal plane using B-mode ultrasound, and the diameter of the brachial artery is measured continuously. A cuff is placed either around the forearm or above the antecubital fossa. After a baseline, resting image is acquired, arterial occlusion is induced by cuff inflation to a pressure above the systolic pressure, typically to at least 50 mmHg above systolic pressure for 5 minutes. When the cuff is released, FMD is calculated as the maximum percent increase in the diameter during hyperemia compared with the baseline diameter.

## 3. Clinical Trials Using FMD

Several studies were reviewed that investigated changes in FMD due to treatment in hypertensive patients. We selected 38 papers published from January 1999 to June 2011 using a PubMed-based search engine. The keywords used in the search were “flow-mediated vasodilation” and “hypertension”, and the original articles were eligible. The appropriateness of all papers identified by the search was confirmed by two experts. When the trial designs of 38 clinical trial papers were classified by the method of Zaza et al. [15], there were 26 randomized controlled trials (RCTs), 5 controlled clinical trials (CCTs), and 7 single-arm trials that were selected (Table 1). The comparisons of multiple antihypertensive drugs were usually done in RCTs or CCTs, and 71 interventions with each antihypertensive drug were investigated in these 38 clinical trial papers. Significant improvement of FMD after antihypertensive treatment was shown in 43 of 71 interventions. 

Medications that block the renin-angiotensin-aldosterone system (RAAS) include angiotensin converting enzyme inhibitors (ACEIs) and angiotensin II receptor blockers (ARBs). These agents are effective in lowering blood pressure and result in organ protection that is effective for clinical conditions such as heart failure [12, 54], chronic kidney disease [12, 55], and diabetes mellitus [12, 56]. As shown in Table 1, most studies have shown that ARBs and ACEIs could improve FMD. One RCT compared the effects of the ACEI enalapril (5–40 mg/day, *n* = 9) and the ARB losartan (50–100 mg/day, *n* = 9) on endothelial function in hypertensive patients [17]. Six months after treatment, FMD improved in both treatment groups compared with baseline levels. In another RCT, patients with HTN were given one of three different ARBs (losartan 50–100 mg/day, *n* = 31; irbesartan 150–300 mg/day, *n* = 30; candesartan 8–16 mg/day, *n* = 31) for 2 months, and the change in FMD was compared among the three groups. ARB therapy significantly improved FMD with no differences among the three groups [23]. The other RCT (cross-over design) also investigated the improvement of FMD during 3 months of treatment with a low or a high dose of ramipril (5 or 10 mg/day, *n* = 46) [28]. While both dosages of ramipril increased FMD, the increase of NO-dependent FMD using N^G^-monomethyl-L-arginine to block NO synthase was greater with the high than with the low dose [28]. In another RCT, treatment with the calcium channel blocker (CCB) amlodipine (5 mg/day, *n* = 22) did not change FMD as much as treatment with the ARB telmisartan (40 mg/day, *n* = 21) for 24 weeks [24]. There were other similar RCTs that compared ACEIs or ARBs with CCBs (perindopril versus amlodipine [34], valsartan versus amlodipine [50], and olmesartan versus amlodipine [52]), and all of these trials showed that ARBs or ACEIs significantly improved FMD compared with CCBs.

The protective effect of CCBs on cardiovascular disease in hypertensive patients has been established in several large-scale clinical trials [12, 57]. However, RCTs which showed improvement of FMD were limited [16, 21, 24, 26, 31, 33, 34, 50, 52].

*β*-blockers have also an established role in cardioprotection [12, 54, 58]. Among various *β*-blockers, nebivolol is a selective *β*-1 adrenergic receptor antagonist and induces endothelial-dependent vasodilation [59]. One RCT compared the effect of nebivolol (5 mg/day, *n* = 20) with atenolol (100 mg/day, *n* = 20), a traditional selective *β*-1 adrenergic receptor blocker without vasodilating properties, on FMD in hypertensive patients [41]. At 4 weeks after treatment, FMD improved more with nebivolol than with atenolol. Furthermore, there were several trials that showed improvement of FMD by nebivolol compared with baseline [36, 43]; however, this drug had less of an effect on FMD than other drug types.

Several RCTs compared three or more types of antihypertensive drugs [21, 23, 25, 29, 49]. One RCT investigated the effects of the ACEI perindopril (2–4 mg/day, *n* = 28), the ARB telmisartan (80–160 mg/day, *n* = 29), the CCB, nifedipine (30–60 mg/day, *n* = 28), the CCB amlodipine (5–10 mg/day, *n* = 28), the *β*-blocker atenolol (50–100 mg/day, *n* = 29), and the *β*-blocker nebivolol (5–10 mg/day, *n* = 28) on FMD [21]. Interestingly, only perindopril significantly improved FMD, whereas all the drugs reduced blood pressure to similar levels.

Furthermore, there was one study that examined the significance of FMD as a prognostic marker of cardiovascular events. Postmenopausal women (*n* = 400) with HTN and impaired FMD received 6 months of antihypertensive therapy (the choice of the antihypertensive drug used was at the discretion of the study investigators) and then were followed-up for a mean period of 67 months. After 6 months of treatment, 250 women showed significantly improved FMD, and this group had significantly fewer cardiovascular events compared with the group without improved FMD (there were no deaths from cardiac causes during the study period) [8].

A prospective study showed the relationship between a low level of FMD and cardiovascular events (although this study did not necessarily have an intervention) [60]. Hypertensive patients (*n* = 172) were divided into a low- and high-FMD group based on the median level of FMD and were followed for 95 months [60]. The incidence of cardiovascular events was 1.4 and 3.1 per 100 patient-years in the low and high groups, respectively. In a Cox proportional hazards analysis, the low-FMD group showed a 2.67-fold increased risk of cardiovascular events [60].

**Figure 1:**
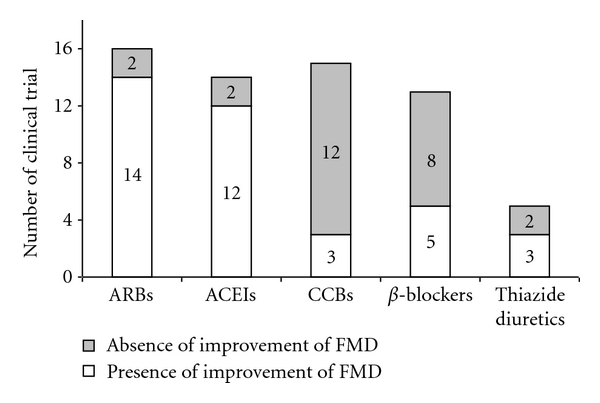
Columns indicate the number of clinical trials that showed the presence or the absence of significant improvement of flow-mediated vasodilation from baseline due to the intervention. FMD: flow-mediated vasodilation; ARBs: Angiotensin II receptor blockers; ACEIs: Angiotensin converting enzyme inhibitors; CCBs: Calcium channel blockers.

Given the overall data, antihypertensive treatment can improve endothelial dysfunction when assessed by FMD. The results of clinical trials showing the effects of different drug types on change in FMD are summarized in Figure 1. More interventions that showed significant improvement of FMD appeared to be found in patients treated with ARBs and ACEIs than those treated with other drug types. However, this is not conclusive, because there has been no single RCT that compared the effects of all drug types on FMD.

Although discrepant results among trials remain to be resolved, they may have been due to differences in the characteristics of patients, the experimental design (i.e., cross-over or parallel group, and the duration of treatment in the intervention), the sample size (relating to the study power) and statistical methodology, and measurement issues (e.g., reproducibility of FMD) [61–63]. A previous study demonstrated that the coefficients of variation of FMD ranged from 6.6 to 10.6% in healthy adults that underwent repeated FMD at intervals of up to 3 months (*n* = 42, mean age = 43) [61]. Patients with atherosclerotic risk factors can also show decreased reproducibility of FMD due to the progression of atherosclerosis over time (e.g., with structural and functional changes of arteries) [62, 63].

## 4. Mechanisms of the Improvement of FMD

Endothelial dysfunction is generally caused by a reduction of endothelium-derived relaxing factors (EDRFs) such as NO, failure of smooth muscle cells to respond to EDRF, activation of the RAAS, and production of vasoconstrictors such as endothelin [1–5]. These factors are also influenced by vascular inflammation associated with oxidative stress [1–5]. However, damaged endothelial cells can be replicated locally and develop into mature endothelial cells with normal function [1, 64, 65]. Circulating endothelial progenitor cells from bone marrow also participate in the repair of the endothelium [1, 64]. The balance between endothelial impairment and repair is a major determinant of endothelial function. Of note, there have been trials that measured biochemical markers such as NO, oxidative stress, and inflammatory markers, in addition to FMD [17, 18, 22, 23, 27, 29, 31, 32, 35, 37, 38, 41, 43, 44, 46, 49, 51–53]. One RCT compared the CCB azelnidipine (16 mg/day) with the CCB benidipine (4 mg/day) in a cross-over design with 8 weeks on each drug [37]. That study examined serum levels of NO and malonyldialdehyde low-density lipoprotein (MDA-LDL) and found that there was no difference in FMD or the levels of NO and MDA-LDL between the two drugs. Another cross-over RCT (*n* = 13, 4 weeks on each drug) compared the effects of losartan (100 mg/day) and atenolol (100 mg/day) on FMD and the levels of 8-isoprostane, a marker of oxidative stress [32]. Losartan, but not atenolol, significantly improved FMD and reduced 8-isoprostane levels [32]. In hypertensive patients treated with zofenopril (15–30 mg/day, *n* = 15), ramipril (2.5–5 mg/day, *n* = 15), or atenolol (50–100 mg/day, *n* = 15) for 8 weeks, there was a similar improvement in FMD, but only zofenopril significantly reduced plasma hydroperoxides, 8-isoprostanes, oxidized-LDL, and adhesion molecules [29]. Other trials have shown that NO, oxidative stress, and inflammatory markers can change in parallel with changes in FMD [17, 18, 22, 23, 27, 29, 32, 35, 38, 41, 43, 46, 51, 52].

Angiotensin II causes vasoconstriction and increases the production of reactive oxygen species (ROS) by activating NADH/NADPH oxidase through the angiotensin II type 1 receptor [66, 67]. ARBs specifically bind to the angiotensin II type 1 receptor and inhibit vasoconstriction, fluid retention, and sympathetic nerve activity by blocking the effects of angiotensin II [68, 69]. Furthermore, ARBs promote the activation of the angiotensin II type 2 receptor involved in NO production [70]. ARBs also decrease oxidative stress within the vessel wall by reducing the production of ROS and increasing NO production, which leads to improved endothelial function.

ACEIs block the RAAS and augment prostaglandins and the kallikrein-kinin system, and these effects lead to a decrease in blood pressure [68, 69]. ACEIs also activate NO production through suppression of the degradation of bradykinin [68, 69]. Whereas ACEIs are different from ARBs in their site of action, both drugs have a common action to inhibit the effects of angiotensin II [68, 69], and most studies showed that these two drug types improved endothelial function.

CCBs have antihypertensive effects that are mediated through the relaxation of vascular smooth muscle cells, and antioxidative effects that are mediated through an increased release of NO [71, 72].

*β*-blockers have scavenging activity for ROS, and these effects are possibly useful in preventing oxidative damage in HTN [73]. However, the improvement of FMD by *β*-blockers was not as great as that observed with other antihypertensive agents. Nebivolol, which is associated with NO-induced vasodilation, improved endothelial function more than other *β*-blockers.

Atherosclerotic risk factors such as diabetes mellitus and dyslipidemia also affect endothelial function, similar to HTN. These risk factors are often observed in patients with HTN. While the effects of these risk factors on FMD should be considered, specific trials for patients with diabetes mellitus or dyslipidemia were not included among the studies cited in the present paper.

## 5. Perspective of Research on FMD in HTN

HTN is a major atherosclerotic risk factor, and antihypertensive treatment protects against future cardiovascular events. The evaluation of endothelial function can be useful in the risk assessment of hypertensive patients treated with antihypertensive drugs. However, larger clinical trials that include morbidity and mortality as outcome variables are needed to further establish FMD as a prognostic marker in hypertensive patients. The influence of other atherosclerotic risk factors and confounding factors (such as obesity, diabetes, cigarette smoking, and dyslipidemia) on FMD in hypertensive patients should also be evaluated in future studies that attempt to establish the prognostic value of FMD. Furthermore, future studies should examine the association between the reduction in blood pressure and the improvement in FMD. In addition, changes in FMD should be compared with sonographical findings of carotid arteries, ankle brachial index, and pulse wave velocity, which have already been widely adopted as noninvasive methods to evaluate atherosclerosis.

## 6. Conclusions

The improvement of FMD in hypertensive patients is expected to slow the progression of atherosclerosis and improve long-term outcomes in these patients. The results of many but not all clinical trials suggest that there is a significant effect of antihypertensive therapy on FMD. ARBs and ACEIs appeared to improve FMD relative to other drug types. Antihypertensive treatment can generally improve endothelial dysfunction as assessed by FMD, although further research is needed.

## Figures and Tables

**Table 1:** Clinical trials on FMD in patients with HTN.

Reference	Patients	Trial design	Intervention	Treatment period	FMD (%)	Notes
Muiesan et al., 1999 [16]	58	Single-arm	An ACEI or a dihydropyridine CCB or a diuretic, in combination with a *β*-blocker	6 months	3.1 ± 3.0 → 6.5 ± 4.5*	—
	10	RCT	Nifedipine	2 months	5.0 ± 6.1 → 9.4 ± 3.9^∗, a^	^ a^ *P* < 0.05 versus Hydrochlorothiazide
	10		Hydrochlorothiazide		5.1 ± 5.2 → 4.6 ± 4.3	

Yavuz et al., 2003 [17]	9	RCT	Enalapril 5–40 mg/day	6 months	8.4 ± 4.5 → 14.0 ± 4.0*	—
	9		Losartan 50–100 mg/day		7.9 ± 3.9 → 12.4 ± 1.9*	

Felmeden et al., 2003 [18]	76	Single-arm	Either Amlodipine and/or Perindopril, or Atenolol and/or Bendroflumethiazide	6 months	4.8 ± 1.3 → 7.3 ± 1.7*	Lipid-lowering treatment (+)

Tezcan et al., 2003 [19]	9	Single-arm	Enalapril 5–40 mg/day	6 months	7.3 ± 3.1 → 16.0 ± 2.9*	—

Munakata et al., 2003 [20]	12	CCT	ACEI (Temocapril/Cirazapril 2/0.5 mg/day)	12 months	12.4 (3.5) → 25.8 (6.3)*	—
	24		CCB (Amlodipine/Benidipine 2.5/4 mg/day)		18.8 (4.4) → 30.0 (5.1)	

Ghiadoni et al., 2003 [21]	28	RCT	Perindopril 2–4 mg/day	6 months	5.1 ± 2.0 → 6.4 ± 2.4^∗, c^	^ c^ *P* < 0.05 versus all other intervention groups
	29		Telmisartan 80–160 mg/day		5.5 ± 2.1 → 5.6 ± 1.9	
	28		Nifedipine 30–60 mg/day		5.2 ± 2.1 → 4.8 ± 1.9	
	28		Amlodipine 5–10 mg/day		5.4 ± 2.0 → 5.1 ± 1.8	
	29		Atenolol 50–100 mg/day		5.5 ± 2.1 → 5.7 ± 1.9	
	28		Nebivolol 5–10 mg/day		5.3 ± 2.2 → 5.6 ± 2.4	

Koh et al., 2004 [22]	47	RCT	Simvastatin 20 mg/day	2 months	4.8 (0.1) → 6.5 (0.1)*	Hypercholesterolemia (+)
			Simvastatin/Losartan 20/100 mg/day		4.7 (0.1) → 7.8 (0.1)^∗, d, e^	^ d^ *P* < 0.05 versus Simvastatin
			Losartan 100 mg/day		4.7 (0.1) → 6.1 (0.1)*	^ e^ *P* < 0.05 versus Losartan

Koh et al., 2004 [23]	31	RCT	Losartan 50–100 mg/day	2 months	4.9 (0.4) → 6.0 (0.4)^∗, f^	^ f^ *P* < 0.05 versus placebo
	30		Irbesartan 150–300 mg/day		4.9 (0.3) → 6.6 (0.3)^∗, f^	No difference among groups
	31		Candesartan 8–16 mg/day		5.0 (0.2) → 6.3(0.2)^∗, f^	

Morimoto et al., 2006 [24]	22	RCT	Amlodipine 5 mg/day	24 weeks	4.2 (0.7) → 3.1 (0.9)	—
	21		Telmisartan 40 mg/day		2.7 (0.8) → 5.7(1.0)^∗, g^	^ g^ *P* < 0.05 versus Amlodipine

Souza-Barbosa et al., 2006 [25]	18	RCT	Hydrochlorothiazide 25–50 mg/day	12 weeks	7.3 ± 2.0 → 12.8 ± 3.1*	No difference among groups
	16		Irbesartan 150 mg/day		7.1 ± 2.8 → 13.0 ± 2.9*	
	14		Quinapril 20 mg/day		7.2 ± 2.8 → 13.2 ± 2.1*	
	15		Irbesartan/Quinapril 150/20 mg/day		7.5 ± 1.9 → 12.8 ± 3.0*	
Mohler et al., 2006 [26]	33	RCT	Amlodipine/Benazepril 5/20–40 mg/day	12 weeks	8.1 [median only] → 10.3*	No difference between both groups
	37		Amlodipine 5–10 mg/day		7.0 [median only] → 8.6*	

Oshima et al., 2006 [27]	20	RCT	Efonidipine 20 mg/day	12 weeks	9.6 ± 3.3 → 9.5 ± 2.9	FMD/NTG ratio was increased
	20		Nifedipine 20 mg/day		9.7 ± 3.0 → 8.0 ± 2.0*	

Ghiadoni et al., 2007 [28]	46	RCT	Ramipril 5 mg/day	3 months	4.6 ± 1.8 → 5.9 ± 2.1*	Comparison of dosage
			Ramipril 10 mg/day		4.6 ± 1.8 → 6.3 ± 2.4*	No difference between dosages

Pasini et al., 2007 [29]	15	RCT	Zofenopril 15–30 mg/day	8 weeks	5.3 ± 1.6 → 6.9 ± 1.7*	—
	15		Ramipril 2.5–5 mg/day		5.4 ± 2.0 → 5.7	The mean after the intervention is estimated by the figure (detailed data not presented)
	15		Atenolol 50–100 mg/day		5.3 ± 1.8 → 5.4	

Buus et al., 2007 [30]	15	RCT	Perindopril 4–8 mg/day	1 year	2.7 (0.3) → 3.4 (0.2)*	No difference between groups
	16		Atenolol 50–100 mg/day		3.3 (0.2) → 4.0 (0.2)*	

Benndorf et al., 2007 [31]	12	RCT	Telmisartan 80 mg/day	6 weeks	5.4 ± 3.3 → 10.9 ± 4.6*	—
	13		Nisoldipine 20 mg/day		6.6 ± 3.3 → 5.9 ± 3.7	
	12		Telmisartan/Nisoldipine 80/10 mg/day		4.5 ± 1.6 → 10.8 ± 4.7*	

Flammer et al., 2007 [32]	13	RCT	Losartan 100 mg/day	4 weeks	2.6 → 3.4 (0.4)^h^	DM (+), The mean of baseline is estimated by the figure (detailed data not presented) ^h^ *P* < 0.05 versus Atenolol
			Atenolol 100 mg/day		2.6 → 2.5 (0.4)	

Morimoto et al., 2007 [33]	25	RCT	Amlodipine 5 mg/day	24 weeks	4.4 (0.8) → 3.2 (0.9)	No difference between groups
	25		Cilnidipine 10 mg/day		4.2 (0.7) → 5.2 (1.0)	

Morimoto et al., 2008 [34]	16	RCT	Amlodipine 5 mg/day	24 weeks	3.0 (0.6) → 2.0 (0.8)	Addition to ARB monotherapy
	16		Perindopril 4 mg/day		2.7 (0.8) → 5.2(0.9)^∗, i^	^ i^ *P* < 0.05 versus Amlodipine

Hirooka et al., 2008 [35]	9	CCT	Valsartan 80–160 mg/day	1 year	5.8 (1.2) → 10.7 (1.4)*	—
	9		Amlodipine 5–10 mg/day		8.6 → 7.7	The mean is estimated by the figure (detailed data not presented)

Korkmaz et al., 2008 [36]	27	CCT	Quinapril 20 mg/day	4 weeks	4.7 ± 3.9 → 5.6 ± 6.1	—
	27		Nebivolol 5 mg/day		3.7 ± 4.2 → 8.5 ± 6.3*	*P* = 0.06 versus Quinapril
Yamada et al., 2008 [37]	21	RCT	Azelnidipine 16 mg/day	8 weeks	Unknown → 4.2 ± 0.7	No difference between groups
			Benidipine 4 mg/day		Unknown → 4.7 ± 0.6	

Farkas et al., 2008 [38]	53	Single-arm	Quinapril 40 mg/day	24 weeks	2.8 ± 1.2 → 8.0 ± 2.5*	Postmenopausal women

Rossi et al., 2008 [39]	180	CCT	Antihypertensive regimens	1 year	2.4 ± 2.2 → 4.8 ± 3.5*	Postmenopausal women with MS
	170	(lifestyle modification or drugs)	4.4 ± 2.5 → 11.5 ± 4.4^∗,j^			Postmenopausal women without MS ^j^ *P* < 0.05 versus the MS group

Jennings et al., 2008 [40]	20	RCT	Lisinopril 10–40 mg/day	1 year	4.4 (1.0) → 7.1 (1.0)*	No difference between groups
	23		Atenolol 25–100 mg/day		4.5 (0.8) → 7.0 (0.8)*	

Pasini et al., 2008 [41]	20	RCT	Nebivolol 5 mg/day	4 weeks	5.9 ± 1.9 → 7.5 ± 2.2^∗, k^	^ k^ *P* < 0.05 versus Atenolol
	20		Atenolol 100 mg/day		5.8 ± 2.1 → 6.1 ± 2.3	

Kosch et al., 2008 [42]	25	RCT	Valsartan 80–160 mg/day	12 weeks	3.5 → 6.1	No difference between groups
	27		Metoprolol 50–100 mg/day		3.9 → 11.2*	The mean is estimated by the figure (detail data not presented)

Merchant et al., 2009 [43]	33	Single-arm	Nebivolol 5–10 mg/day	8 weeks	3.4 ± 0.4 → 11.0 ± 1.3*	Obesity (+)

Jung et al., 2009 [44]	39	Single-arm	Telmisartan 80 mg/day	8 weeks	7.6 ± 3.5 → 9.0 ± 2.8*	—

Ubaid-Girioli et al., 2009 [45]	39	CCT	Spironolactone 25 mg/day	6 months	7 → 10*	Poorly controlled HTN Added to multiple agents The median is estimated by the figure (detailed data not presented)

Yamanari et al., 2009 [46]	14	RCT	Chlortalidone 25 mg/day	16 weeks	8.1 ± 2.0 → 6.0 ± 1.5*	Poorly controlled HTN
	14		Spironolactone 25 mg/day		8.1 ± 2.0 → 8.8 ± 2.7^l^	Addition to both Amlodipine and Candesartan ^l ^ *P* < 0.05 versus Chlortalidone
Perrone-Filardi et al., 2009 [47]	13	RCT	Candesartan 16 mg/day	2 months	5.2 ± 1.6 → 7.1 ± 2.6^∗, m^	Coronary artery disease (+), added to *β*-blocker ^m^ *P* < 0.05 versus placebo

Ghiadoni et al., 2009 [48]	31	RCT	Perindopril/Indapamide 2–4/0.625–1.25 mg/day	24 weeks	5.0 ± 2.1 → 6.0 ± 1.7*	No difference between groups
	31		Atenolol 50–100 mg/day		5.1 ± 1.8 → 5.5 ± 1.8	

Brandão et al., 2010 [49]	29	RCT	Perindopril 4 mg/day	12 weeks	7.3 [4.3–10.3] → 9.3 [6.3–11.2]*	No difference among groups
	33		Hydrochlorothiazide 25 mg/day		8.3 [5.8–10.1] → 9.0 [7.2–11.7]*	
	32		Indapamide 1.5 mg/day		6.7 [5.0–10.2] → 9.2 [6.7–12.3]*	

Yilmaz et al., 2010 [50]	37	RCT	Amlodipine 10 mg/day	12 weeks	6.7 [*5.5–8.3*] → 7.5 [5.0–8.7]*	DM (+), CKD stage 1
	35		Valsartan 160 mg/day		6.3 [5.5–8.0] → 7.8 [6.0–9.3]^∗,n^	^ n^ *P* < 0.05 versus Amlodipine
	36		Valsartan/Amlodipine 160/10 mg/day		6.4 [5.5–7.3] → 8.0[6.9–9.3]^∗, n^	

Wago et al., 2010 [51]	35	Single-arm	Telmisartan 40 mg/day	1 year	4.0 ± 2.2 → 5.9 ± 2.2*	DM (+)

Takiguchi et al., 2011 [52]	31	RCT	Olmesartan 20–40 mg/day	12 weeks	3.9 ± 3.0 → 6.1 ± 3.1^∗, o^	^ o^ *P* < 0.05 versus Amlodipine
			Amlodipine 5–10 mg/day		3.9 ± 3.0 → 4.6 ± 2.6	

Heffernan et al., 2011 [53]	24	RCT	Atenolol 50 mg/day	4 weeks	8.4 (1.1) → 9.1 (1.4)	No difference between groups
			Metoprolol 50 mg/day		8.4 (1.1) → 10.3 (1.4)	

FMD: flow-mediated vasodilation; HTN: hypertension; ACEI: angiotensin converting enzyme inhibitor; CCB: calcium channel blocker; RCT: randomized controlled trial; COX-2: cyclooxygenase-2; CCT: controlled clinical trial; DM: diabetes mellitus; PDE5-I: phosphodiesterase type 5 inhibitor; NTG: nitroglycerin-induced vasodilation; ARB: angiotensin II receptor blocker; MS: metabolic syndrome; CKD: chronic kidney disease. Data are presented as the mean ± standard deviation, mean (standard error), or median (interquartile range or total *range*). *P < 0.05 compared with before intervention.
